# Repeatability of measures of behavioral organization over two years in captive infant rhesus macaques, *Macaca mulatta*

**DOI:** 10.1002/ajp.23591

**Published:** 2024-01-11

**Authors:** Alexander J. Pritchard, John P. Capitanio, Laura Del Rosso, Brenda McCowan, Jessica J. Vandeleest

**Affiliations:** 1 Neuroscience and Behavior Unit, California National Primate Research Center, University of California, Davis, California, USA; 2 Department of Population Health and Reproduction, School of Veterinary Medicine, University of California, Davis, California, USA

**Keywords:** Biobehavioral Assessment, development, infants and juveniles, personality, temperament

## Abstract

Individual differences of infant temperament have been associated with future health outcomes that provide explanatory power beyond adult personality. Despite the importance of such a metric, our developmental understanding of personality-like traits is poor. Therefore, we examined whether young primates show consistency in personality traits throughout development. We replicated a Biobehavioral Assessment (BBA) at three time periods: 3–4 months, 1 year, and 2 years of age in 47 rhesus macaque (*Macaca mulatta*) subjects from large mixed-sex outdoor social housing units at the California National Primate Research Center. We report results for tests focused on responses and adaptation to the temporary separation and relocation, responses to a threatening stimulus, and ratings of overall temperament. We found consistently repeatable associations in measures of Emotionality; these associations were stronger in males, but also present in females, and broadly consistent between Years 1 and 2. We also explored whether behavioral responses to this experimental relocation might be influenced by their experience being relocated for other reasons (i.e., hospitalizations) as individuals’ responses might be influenced by similar experiences to the BBA procedure. Only locomotion, during one of the assessments, was associated with past hospitalization events. Overall, repeatability in Emotionality-associated behaviors was evident across the 2 years, in both sexes. We did, however, find evidence of the emergence of sex differences via differentiated expression of behavioral responses during the BBA. We emphasize that there is likely contextual nuance in the use of these BBA factor-associated behaviors. Further research is required to determine whether and how shifts occur in underlying factor structure and the expression of associated behaviors.

## Graphical Abstract

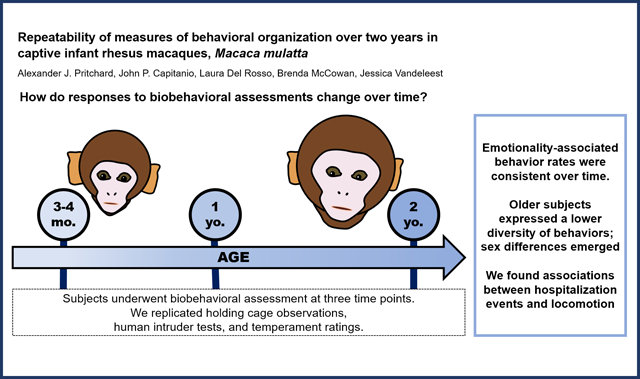

## 1 | INTRODUCTION

Development of personality-like traits in nonhumans is an understudied phenomenon (Cabrera et al., 2021). This gap is problematic given that infant personality scores have been associated with future health outcomes (as reviewed in: Capitanio, 2021; Hampson & Vollrath, 2012), and provide explanatory power distinct from adult personality measures (Wright & Jackson, 2022). The literature using human subjects poorly represents infant personality as a continuum to adult personality and the associated health outcomes (as reviewed in: Caspi et al., 2005). In determining how latent processes shift over time, this gap is problematic for developing an understanding of how robust personality-associated measures are over time, and, ultimately, how personality traits mechanistically exert changes on health outcomes (Simpson, Robinson, et al., 2019).

To extend the link between infant assessments of individual differences and later growth periods, we replicated an established infant test for individual differences, the Biobehavioral Assessment (BBA), at two later time periods. The BBA program has been active at the California National Primate Research Center (CNPRC) since 2000, quantifying individual differences in over 5000 infant rhesus macaques (*Macaca mulatta*) (reviewed in: Capitanio, 2017, 2021). Rhesus macaques are important translational health models due to the demonstrated influence of individual differences on health outcomes (Capitanio, 2011, 2021; Elfenbein et al., 2016; Maninger et al., 2003)—an understanding of how infant temperament is bridged to adult personality is a key next step. The robust BBA data set facilitates a powerful foundation on which to build our understanding of personality development through the replication of BBA at older time periods. Thus, in this present study, we have defined personality factors isolated from the initial BBA sampling point (Golub et al., 2009; Gottlieb & Capitanio, 2013). The BBA program involves a battery of tests on infants, which most relevant here include behavioral reaction and adaptation to a challenge, response to human intruder tests, as well as temperament ratings. The personality factors isolated using these tests include: Activity and Emotionality from holding cage observations on Day 1 (behavioral reaction to a challenge) and Day 2 (adaptation to a challenge) (Golub et al., 2009); Confident, Gentle, Nervous, and Vigilant from temperament ratings (Golub et al., 2009); and Activity, Aggression, Displacement, and Emotionality from human intruder tests (Gottlieb & Capitanio, 2013). BBA is conducted on infants at 3–4 months of age, important as this age period reflects the early stages of the dietary and social transitions that are associated with the onset of weaning (Capitanio, 2021).

Heretofore, BBA has been primarily used to assess biobehavioral organization of 3–4 month-olds; in this exploratory study, we aimed to determine whether biobehavioral organization shows individual consistency from infancy to 2 years of age. Thus, an abbreviated form of the established infant BBA testing paradigm was repeated at 1 and 2 years of age. The importance of these outcomes is threefold: to establish a baseline understanding of the continuity between early measures of BBA factors and later factor-associated measures, to catalog mean-level developmental trajectories, and to facilitate testing in older individuals for similar traits. We had three broad hypotheses, that: (1) individuals would show shared growth trajectories on measures of biobehavioral organization, exhibiting within-individual consistency, (2) sex differences would emerge as individuals mature, and (3) individuals’ life experiences during development would alter their behavioral responses to each test.

We hypothesized that growth trajectories would be observable, such that BBA factors would shift over time—whereby individuals would exhibit consistency in relative rank position compared with their peers, but mean or median scores shift over time. Such a trend is commonly reported. For instance, focal observations of free-ranging rhesus macaques reported steady age-related declines in fear grimaces, social withdrawals, and resting—which the authors linked to a fearfulness factor—and increases in an aggression factor (von Borell et al., 2016). Only the fearfulness factor showed repeatability across the 7-year study period, however. Similarly, Stevenson-Hinde et al. (1980) reported an increase in median Confident scores over the first 3 years of life in captive infant rhesus macaques based on ratings from colony observations, which were significantly consistent over time. Finally, Clarke and Snipes (1998) also reported declines in fearfulness during peer group home cage observations, but increases in activity and excitability, as individuals aged. Thus, we expected that infants would decrease in fear-associated behaviors and become more active as they mature.

Typically, at 3–4 months of age, sex differences are not observed in the BBA outcomes (Golub et al., 2009). This is somewhat unsurprising, because sex differences in behavioral expression often arise later at 1–2 years of age in infant rhesus macaques (Kulik et al., 2015a, 2015b; Lovejoy & Wallen, 1988), with menarche occurring in captivity at around 2.5 years (Zehr et al., 2005). Personality development studies in nonhumans, however, have reported the emergence of sex differences from somewhere between 3 months and sexual maturation (Clarke & Snipes, 1998; von Borell et al., 2016). Therefore, we examined male and female subjects distinctly as sex differences might emerge during later test points. Importantly, sex differences may not emerge evenly across all behavioral types, even by 2 years of age (Wallen, 1996). For example, at 1 year of age, males in captivity were more likely to engage in playful behaviors (Lovejoy & Wallen, 1988), though these differences in play likely emerge even earlier in free-ranging macaques (Kulik et al., 2015b). Grooming rates, instead, have been reported to show a peak sex difference at 2 years of age despite diverging earlier (Kulik et al., 2015b). Similarly, there are sex differences in the age at which rates of initiated aggression peak despite similar prepubertal rates: males’ peak aggression occurred at 2 years of age, while females peaked at 3 years (Kulik et al., 2015a). In humans, personality development differences between boys and girls are understudied in children under 10 (Slobodskaya, 2021). As girls approach adolescence, however, they score higher on Neuroticism (Slobodskaya, 2021) such that, by the age of 14, females scored higher in Neuroticism relative to males (De Bolle et al., 2015). Personality sex differences are already present at the age of 12 years, with adolescent females scoring higher on Conscientiousness and Openness relative to males (De Bolle et al., 2015). In humans, menarche often occurs at an age of 12–13.5 years across cultures (Karapanou & Papadimitriou, 2010). De Bolle et al. (2015) reported that personality sex differences, however, were not pronounced (most were between 0.25 and 0.50 of the SD) even though they likely persist into adulthood. Indeed studies generally report that mean-level differences between boys and girls are slight, albeit with higher scores in Activity for boys, relative to girls; and, higher scores in Agreeableness, Conscientiousness, as well as Effortful Control for girls, relative to boys (Slobodskaya, 2021).

While our primary focus is on the effects of development, prior research supports that personal experience during growth can also alter the development of personality traits in rhesus macaques. For instance, rearing condition can alter personality-associated measures and ratings (Capitanio et al., 2006; Clarke & Snipes, 1998). Indeed, evidence indicates that increased handling and engagement events from human givers across the first 12 weeks of life were associated with heightened novelty-seeking behavior relative to a control group that underwent less handling, in 48 captive rhesus macaques (Simpson, Sclafani, et al., 2019). Our subjects were reared in the same large mixed-sex groups, however, we acknowledge other experiences may influence outcomes. Therefore, we also examined whether hospitalization events covaried with our measures. We included hospitalization because the conditions of capture, within-center transport, and housing are comparable to the experience infants would go through during BBA. Thus, we expected that repeat hospitalizations may result in desensitization to aspects of the BBA and potentially reduce emotionality-associated measures.

We used replicated BBA events to determine whether individuals would exhibit within-individual stability via rank-consistency in rates and proportions of behaviors associated with BBA factors. We expected that sex differences would emerge within these metrics as individuals mature, with greater divergence between the sexes for rates and proportions of BBA associated behaviors. Additionally, we explored whether experiences with hospitalization during development would be associated with rates and proportions of the same BBA associated behaviors across years.

## 2 | METHODS

### 2.1 | Study subjects

We studied 47 rhesus macaques (*Macaca mulatta*) born into a single, outdoor, half-acre, mixed-sex housing unit across 2 years (2016 and 2017) at the CNPRC. This group contained between 120 and 156 animals (mean = 133.5) of all ages across the four study years (2016–2019) with a mean female:male sex ratio of 2.05. The majority of the infant subjects born in this housing unit during the study’s recruitment years (2016 and 2017) were assessed in the infant BBA program, with a single exception due to an unavoidable management event; mean age of infant sampling was 102.51 ± 8.33SD days. Our subjects underwent two addition replicates for a subset of the BBA tests at 1 and 2 years of age (mean ages, respectively, 361.56 ± 10.28SD days and 724.98 ± 11.51SD days). For this study, we replicated the holding cage observations and human intruder tests, in both years, and the temperament ratings in Year 1. Overall, 47 individuals were enrolled in the study in Year 1, with 45 resampled in Year 2. In the Year 1 tests, we had 27 females and 20 males; one of these subjects (a female) was excused from testing in the afternoon of Day 1 due to unavoidable management events and missed Day 2 of the holding cage observations. For the Year 2 data, we had 24 females and 21 males; two of these subjects (one male and one female) were excused from testing in the afternoon of Day 1 due to unavoidable management events and missed Day 2 of the holding cage observations. Additionally, one male subject was not sampled in the infant BBA; his data were used only in the Year 1 versus Year 2 comparisons. When possible, we included all available subjects for the relevant analyses.

Our protocols were approved by the Institutional Animal Care and Use Committee at the University of California, Davis, and also adhered to the Principles for the Ethical Treatment of Non-Human Primates, set by the American Society of Primatologists.

### 2.2 | Brief description of BBA

The BBA program was initiated at CNPRC in 2000. Although the BBA program tests are detailed elsewhere, we have included brief descriptions of each test that we repeated at Years 1 and 2 (Y1 and Y2) for clarity. In the ongoing program, individuals, who are tested in groups of five to eight animals at a time, are temporarily separated from their mothers and social group, typically when they are between 90 and 120 days of age, and are relocated to an indoor housing room for 25 h during which they undergo several tests (reviewed in: Capitanio, 2017, 2021).

The holding cage observations were conducted by an observer who sat 2.6 m away and coded behaviors real-time at the holding cage (60 × 65 × 79 cm, Lab Products, Inc.) for 5 min on Day 1 (d1) and d2 of the BBA (Golub et al., 2009) using focal animal sampling conducted in a randomized order. These periods of observation reflect the initial behavioral response to social separation (d1) and adaptation to the testing environment (d2). Using exploratory and confirmatory factor analyses, prior work found two factors, using 11 of the 32 coded behaviors: *Activity*, composed of proportions of time hanging (negatively loaded) and locomoting, as well as rates of exploring, crouching, drinking, and eating; and *Emotionality*, composed of rates of lip-smacking vocalizations, self-scratching, threatening, barking vocalizations, and cooing vocalizations (Golub et al., 2009). Scales were formed for Activity and Emotionality for d1, and again for d2.

The human intruder test was conducted 5 h after the initiation of the BBA with subjects relocated into a testing cage (38.7 × 52.1 × 47.0 cm) in an adjacent room (Gottlieb & Capitanio, 2013). The human intruder test focused on responses of the subjects to a graded series of challenges presented by the BBA technician. The test lasted for 4 min, with a human experimenter changing her position (in profile or staring) and distance (1 m and 0.3 m) to the animal subject each minute. The experiment was video recorded and coded for the presence of 31 behaviors. Exploratory and confirmatory factor analyses on data from the infants found four factors from 12 behaviors: *Activity*, composed of proportions of time active, as well as rates of exploring and cage shaking; *Emotionality*, composed of rates of convulsive jerks, cooing vocalizations, grimacing, and self-clasping; *Aggression*, composed of rates of barking vocalizations, threatening, and other vocalizations (not cooing, lip-smacking, or barking); and *Displacement*, composed of rates of tooth grinding and yawning (Gottlieb & Capitanio, 2013). Here we use Displacement as shorthand for displacement activities or behaviors as measures of anxiety, frustration, or uncertainty (e.g., Maestripieri et al., 1992).

Finally, temperament ratings were completed at the end of the 25 h for each subject by the technician that interacted with the animals across the entire testing period. Surveys included 16 adjectives that were rated on a 1–7 Likert scale (Golub et al., 2009). Exploratory and confirmatory factor analyses revealed four factors from 14 inclusively clustering items: Confident composed of active, bold, *confident*, curious, and playful items; *Gentle* composed of calm, curious, flexible, and gentle items; *Nervous* composed of calm (negatively loaded), confident (negatively loaded), fearful, nervous, and timid items; and *Vigilant* composed of depressed (negatively loaded), tense (negatively loaded), timid (negatively loaded), and vigilant items (Golub et al., 2009).

### 2.3 | BBA replicates for the present study

We replicated the BBA procedure when the infant subjects reached one and 2 years of age. These BBA tests are conducted annually for many infants by the same staff and, as a consequence, were functionally similar across the replicate runs with the exception of several minor modifications. Generally modifications included: removal of one of the assessments from the original BBA program for 2-year-olds, and adjusting cage sizing as well as layout to accommodate the larger juveniles (i.e., human intruder tests were conducted in the holding cages for the 2-year-olds). Specifically, (1) after the holding cage observations on d2, but before the temperament ratings, infants typically received an adrenocorticotropic hormone injection as a part of a cortisol reactivity assessment. Subjects at 1 and 2 years of age did not receive this injection. (2) At the end of the BBA assessment for 2-year-olds, we substituted a food retrieval test of a familiar-but-desirable food (a grape) instead of completing the temperament ratings. This change was implemented, in part, because the reduction in the number of tests for the 2-year-olds (detailed above) reduced the BBA technician’s familiarity with the subjects. The food retrieval test has been demonstrated as an effective measure of an inhibited temperament, a composite measure of BBA holding cage Emotionality and Activity; low Activity scores and low Emotionality across both days of BBA were predictive of later refusal of an offered food item as adults (with an average age over 4) (Fox et al., 2021). Overall, the testing schedule remained the same such that subjects remained in their temporary living cages during time blocks that omitted tests would occupy. Some additional behaviors were coded at Years 1 and 2, but not included in these analyses. Holding cage sizes (i.e., standard laboratory indoor caging) were not adjusted as individuals aged and, thus, subjects occupied a greater proportion of the cage as they aged; though perch bars were added for Year 2 individuals. For 2-year olds, human intruder testing was conducted in the holding cages to accommodate their larger size.

### 2.4 | Analysis

#### 2.4.1 | Consistency with BBA factor scores across development

Due to the robust sample size of the original BBA factor analyses (Capitanio, 2017), we retained the infants’ original BBA factor scores as a baseline comparison for the later BBA replicates. We used Spearman’s rank correlations to determine whether proportions and rates of behavioral responses to the holding cage observations and human intruder tests in Y1 and Y2 were consistently associated with the original BBA factor scores. We limited the behaviors examined in Y1 and Y2 to the behaviors comprising the factor scores during the BBA infancy events. We acknowledge the challenge of interpreting numerous correlation coefficients. Thus, rather than assess all coded behaviors across the BBA, we focused on behaviors identified through BBA that reliably reflected underlying latent traits as grounds for a more hypothesis-driven approach. We also were conservative in assessing consistency by focusing on behaviors that had moderate correlations across all years, to limit interpretations that would be drawn from a single moderate correlation. We set a moderate effect size (coefficient) cut-off of 0.30 (Cohen, 1988), a reasonable expectation given nonhuman behavior repeatability estimates centered around 0.35 in a large meta-analysis, with reduced estimates from studies having captive subjects or large time intervals between samples (Bell et al., 2009). Indeed, 0.20 has been posited as “typical” in research on human individual differences (Gignac & Szodorai, 2016). We follow recommendations by Althouse (2016) to report unadjusted *p* values—partly due to the exploratory nature of this work (Althouse, 2016), but also because we have functionally penalized our estimates via expectations of moderate and repeatable effect sizes.

The temperament ratings are on a Likert scale, thus raw ratings were difficult to compare with the original BBA temperament factors. Therefore, we created composite sum scores using the relevant items from the original BBA factors. We scaled all items then reverse scored items that negatively loaded on each relevant factor, before combining them into the composite score. Composite scores do not fully represent factor scores, but provide a better score approximation than the raw Likert scale ratings.

#### 2.4.2 | Behavioral consistency between Years 1 and 2

To assess individual consistency between the Y1 and Y2 replicates, we examined whether the rates and proportions of observed behaviors were Spearman rank-correlated.

#### 2.4.3 | Associations of BBA behaviors with hospitalizations

The infant BBA is typically the first infant–mother separation/relocation experience for infants in the indoor laboratory setting. The Y1 and Y2 subjects, however, may experience additional similar events, for example, due to hospital treatments, or other colony related management procedures. These experiences could alter perceptions of the novelty of the testing environment and individual responses to the challenge of social separation and relocation. Therefore, we examined whether the number of individual hospitalization events and total days in the hospital leading up to the annual testing were correlated (Spearman’s rank) with behavioral rates and proportions at the Y1 and Y2 assessments.

## 3 | RESULTS

### 3.1 | Assessing representative subsampling

Our subjects represent a smaller subset of the total infants sampled each given year in the BBA program. As BBA scores are *z*-scored within their birth year, we can examine the distribution of the original BBA scores for our subjects to gain insight as to whether we have a representative subsample for each set of factor scores. Representative samples should have a mean of approximately zero, and an SD approximating 1. Our BBA scores were representative of the two holding cage observation factors across the 2 days (Activity D1: *μ* = 0.02, SD = 1.06, D2: *μ* = 0.13, SD = 1.00; Emotionality D1: *μ* = 0.23, SD = 1.00, D2: *μ* = 0.11, SD = 1.15) and the four temperament factors (Confident *μ* = 0.04, SD = 1.04; Gentle *μ* = −0.11, SD = 1.06; Nervous *μ* = 0.15, SD = 1.02; Vigilant *μ* = 0.06, SD = 1.01). Human intruder test *z*-scores in our subset, however, potentially underrepresented the variation of Displacement and Aggression in the source population, as indicated by smaller standard deviations (Activity *μ* = −0.12, SD = 0.86; Aggression *μ* = −0.08, SD = 0.78; Displacement *μ* = −0.24, SD = 0.67; Emotionality *μ* = −0.12, SD = 1.11).

### 3.2 | Consistency with BBA factor scores across development

As individuals aged, they generally expressed lower rates or proportions of all of the BBA-associated behaviors (Supporting Information: Figures S1 & S2) in both the human intruder test and holding cage observations. The median percent change of each BBA-associated behaviors across tests comparing means between infancy and Y2 was −18.36%, with the largest change observed in cooing during D1 holding cage observations (−414.52%). Of the BBA-associated behaviors, the only increase was seen in rates of lip-smacking during D2 holding cage observations (00.23%). Instead, many of the subjects in Y2 spent much more time sitting (Supporting Information: Figure S3) (median change in proportion of time sitting from infancy to Y2 across tests was 35.03%).

We assessed whether individuals exhibited shared growth trajectories on measures of behavioral organization (hypothesis 1), and whether sex differences emerged as individuals mature (hypothesis 2) by examining individual rank consistency of behavioral measures, within each of the sexes. Here, we report these results organized by the original factor names, and detail any sex differences throughout (Tables 1 & 2). We use terminological distinction of weak, moderate, and high correlations for values < 0.30, ≥0.30, and ≥0.50, respectively, following Cohen (1988).

Broadly, we observed limited consistency in Activity-associated behaviors with the BBA scores (holding cage observation mean |*ρ* | = 0.17 ± 0.15SD; human intruder test mean |*ρ* | = 0.20 ± 0.21SD). We observed higher overall consistency in Emotionality-associated behaviors with the BBA scores (holding cage observation mean |*ρ* | = 0.20 ± 0.27SD; human intruder test mean |*ρ* | = 0.35 ± 0.18SD), with consistently moderate correlations exhibited for a subset of behaviors (detailed below). Although several Aggression- and Displacement-associated behaviors in the human intruder test exhibited moderate-to-high associations with BBA scores across both years (Aggression mean |*ρ* | = 0.31 ± 0.22SD; Displacement mean |*ρ* | = 0.42 ± 0.17SD), these summary statistics do not reflect the fact that there was a large reduction in the expression of BBA-associated behaviors for Y2 juveniles (Table 2). Temperament composite scores exhibited moderate correlations for a subset of the BBA factors (Nervous *ρ* = 0.49; Confident *ρ* = 0.36), but the generalizability of these correlations were difficult to assess given the single replicate.

### 3.3 | Activity

Very few BBA Activity-associated behaviors showed consistency with BBA scores across the 2 years for males or females (Table 1). There was a lack of concordance between which behaviors correlated with infant BBA scores from Y1 and Y2, emphasizing the lack of consistency. The majority of activity-associated behaviors were expressed across years in the holding cage observations, but a subset of behaviors were not expressed in the human intruder test (Table 2).

Activity BBA scores in the holding cage observations for females (Table 1) had moderate negative correlations on d1 with Y2 rates of eating (*ρ* = −0.44), and high positive correlations with Y2 rates of exploration (*ρ* = 0.53). The d2 Activity scores, however, showed a high and moderate negative correlation, respectively, with Y1 proportion of time hanging (*ρ* = −0.52) and Y2 rates of eating (*ρ* = −0.31). Activity BBA scores in the holding cage observations for males (Table 1) showed moderate positive correlations with rates of Y1 exploration across both days (d1 *ρ* = 0.35; d2 *ρ* = 0.32). Proportions of time hanging in d1 (*ρ* = −0.44) and rates of eating in d2 (*ρ* = −0.42) showed moderate negative correlations with Activity BBA scores in Y1 and Y2, respectively.

Activity BBA scores in the human intruder tests, for females (Table 2), were moderately positively correlated with Y1 exploration rate (*ρ* = 0.49) and activity proportion (*ρ* = 0.46). Y2 female behaviors and all of the male behaviors across both years were not associated with BBA Activity scores. This lack of Y2 correlations is partly because our subjects did not express the Activity-associated behaviors of exploration and cage shaking in the Y2 assessments.

### 3.4 | Emotionality

A subset of the BBA-associated behaviors showed moderate-to-high consistency for males and, to a lesser extent, females (Tables 1 & 2). The strength of these relationships were generally decreased from infancy to Y2, relative to correlations from infancy to Y1. All behaviors were expressed across years in the holding cage observations (Table 1), but a subset of behaviors were not expressed in the human intruder test (Table 2).

Emotionality BBA scores for d1 in the holding cage observations for females (Table 1), had high positive correlations with Y1 rates of cooing (*ρ* = 0.52), moderate positive correlations with rates of lip- smacking (*ρ* = 0.40), and moderate negative correlations with Y1 threat rates (*ρ* = −0.30); Y2 behaviors did not show any correlation with the scores in infancy, though threat rates were only marginally weaker in Y2 (*ρ* = −0.24), as compared with Y1. The d2 infant Emotionality scores, however, showed high positive correlations with rates of Y1 lip-smacking (*ρ* = 0.60) and Y1 scratching (*ρ* = 0.56), and moderate positive correlations with Y1 rates of barking (*ρ* = 0.49) and cooing (*ρ* = 0.40), for both years.

Infant emotionality BBA scores in males (Table 1), had moderate-to-high positive correlations with d1 rates of cooing (Y1 *ρ* = 0.76; Y2 *ρ* = 0.52) and barking across both years (Y1 *ρ* = 0.46; Y2 *ρ* = 0.68), as well as lip-smacking (*ρ* = 0.63) and threat rates in Y1 (*ρ* = 0.38). The correlation of lip-smacking in Y2 on d1 were lower than 0.30 (*ρ* = 0.25). The d2 infant Emotionality scores, however, showed no correlations with behaviors across later years that exceeded the threshold of |0.30|, except for a moderate negative correlation with Y1 threat rates (*ρ* = −0.45).

Emotionality BBA scores in the human intruder tests (Table 2), were moderately correlated with Y1 grimace rates for both sexes (male *ρ* = 0.43; female *ρ* = 0.34) and were also moderately correlated with cooing rates in males across both years (Y1 *ρ* = 0.49; Y2 *ρ* = 0.32). These positive correlations were challenging to interpret, however, given they were driven by a few outliers. The lack of Y2 correlations is partly because our subjects did not express the Emotionality-associated behaviors of grimacing and self-clasping in the Y2 assessments. Convulsive jerks were not expressed by any of our juvenile subjects and, thus, are not included in Table 2.

### 3.5 | Aggression

Aggression BBA scores in the human intruder tests (Table 2) were positively correlated with the Y1 rates of barking, highly in males (*ρ* = 0.66) and moderately in females (*ρ* = 0.37). In females, we also observed moderate positive correlations with Y1 rates of threats (*ρ* = 0.39). Our subjects did not express any of the Aggression-associated behaviors in the Y2 assessments.

### 3.6 | Displacement

Displacement BBA scores in the human intruder tests (Table 2) had moderate-to-high positive correlations with Y1 rates of tooth grinding in both sexes (females *ρ* = 0.58; males *ρ* = 0.61) and, for females, Y1 yawning (*ρ* = 0.44). These associations, however, were driven by a handful of individuals. Our subjects did not express any of the Displacement-associated behaviors in the Y2 assessments.

### 3.7 | Temperament factors

For the temperament ratings, cross-correlations varied between the items within each composite score (Supporting Information: Figure S4). The absolute value averages of these cross-correlations were moderate for the confident composite (mean *ρ* = 0.62), moderate-to-low for the vigilant (mean *ρ* = 0.40) and gentle composites (mean *ρ* = 0.34), but low for the nervous composite (mean *ρ* = 0.16).

In female subjects, Nervous BBA scores showed a moderate positive correlation (*ρ* = 0.49) with the corresponding Y1 composite scores. Confident BBA scores showed moderate correlations with the corresponding composite scores (*ρ* = 0.36), while Gentle and Vigilant factor scores were weakly associated (*ρ* = 0.29 and 0.19 respectively) with the corresponding composite scores.

In male subjects, however, we observed weak relationships between the Y1 composite and BBA factor scores (Confident *ρ* = 0.10; Gentle *ρ* = −0.24; Nervous *ρ* = −0.05; Vigilant *ρ* = 0.15).

### 3.8 | Behavioral consistency between Years 1 and 2

We limited our focus to behaviors that exceeded a correlation cut-off with the BBA factor scores (Table 3) as our primary interest was in isolating behaviors that had consistency across infant–juvenile development. We present the remaining BBA-associated behaviors in the supplementary materials (Supporting Information: Table S3).

### 3.9 | Activity-associated behaviors

Activity-associated measures showed poor consistency across the 2 years in the holding cage observations (Table 3). Exploratory behaviors in d1 were moderately correlated across the 2 years (females *ρ* = 0.46; males *ρ* = 0.44). Exploratory behaviors in d2 were moderately correlated across both years for females (*ρ* = 0.42), but not males (*ρ* = 0.15). Hanging was not consistent across years (*ρ* ≤ |0.22 | ), due to low proportions of hanging in Y2. With the exception of d1 rates in males (*ρ* = 0.46), eating rates were not correlated across the years (*ρ* ≤ 0.18).

The Activity-associated measure of proportion of activity in the human intruder test was poorly correlated across the years, for both sexes (*ρ* ≤ 0.09).

### 3.10 | Emotionality-associated behaviors

Emotionality-associated measures showed moderate consistency across the 2 years in the holding cage observations (Table 3). Cooing was moderately correlated for both sexes across both days (mean *ρ* = 0.61 ± 0.10SD). Across both days, barking was strongly correlated for females (d1 *ρ* = 0.62; d2 *ρ* = 0.64) and moderately correlated for males (d1 *ρ* = 0.37; d2 *ρ* = 0.43). Lip-smacking was highly-to-moderately correlated in the male data set (d1 *ρ* = 0.52; d2 *ρ* = 0.33), but for d1 in females (*ρ* = 0.45), not d2 (*ρ* = 0.20). Threat rates had moderate-to-high correlations for females across both years (d1 *ρ* = 0.45; d2 *ρ* = 0.72), and were correlated males in d2 (*ρ* = 0.35), but not d1 (*ρ* = −0.08). Self-scratching on d1 was highly correlated between the 2 years for both sexes (females *ρ* = 0.76; males *ρ* = 0.65), but this was not evident in the d2 data set (*ρ* ≤ 0.19).

The Emotionality-associated measure of cooing in the human intruder test showed a moderate correlation across the years in males (*ρ* = 0.54), but not in females (*ρ* = 0.04).

### 3.11 | Associations of BBA behaviors with hospitalizations

To assess whether individuals’ life experiences during development would alter their behavioral responses to each test (hypothesis 3), we examined whether prior hospitalizations were associated with behavioral measures during holding cage observations across the BBA replicate events. Hospitalization events and cumulative days of hospitalization were correlated at the Y1 (*r*_45_ = 0.86) and Y2 (*r*_43_ = 0.76) testing events, such that individuals that were hospitalized more frequently also spent more cumulative days in the hospital. Similarly, correlations with the BBA behavioral measures were similar across the two metrics. Thus, we only report results using hospitalization events. Hospitalization events did not markedly differ between the two sexes in Y1 (males = 2.25 M ± 1.59; females = 2.44 M ± 1.55), though females had a slight increase in cumulative hospitalizations relative to males in Y2 (females = 3.83 M ± 1.63; males = 3.43 M ± 1.94) (Supporting Information: Figure S5).

Hospitalization events were moderately-to-highly correlated (Table 4) with proportion of time locomoting during the holding cage observations (*ρ* = 0.34 M ± 0.26SD): for males, this was consistent in d1 across both years (Y1 *ρ* = 0.44; Y2 *ρ* = 0.46), but only for Y1 in d2 (*ρ* = 0.81); in females, for Y1 d2 (*ρ* = 0.38) and Y2 d1 (*ρ* = 0.38). Rates of barking were moderately correlated with hospitalization events for males in d1 across both years (Y1 *ρ* = 0.44; Y2 *ρ* = 0.33). Rates of crouching were also weakly-to-moderately negatively correlated with hospitalization events for females in Y1 across both days (d1 *ρ* = −0.23; d2 *ρ* = −0.31).

## 4 | DISCUSSION

We explored our data with three predictions in mind: (1) that individuals would show shared growth trajectories on measures of biobehavioral organization, exhibiting within-individual consistency in BBA-associated behaviors; (2) that sex differences would emerge as individuals mature; and, (3) that certain types of individuals’ life experiences during development would alter their behavioral responses to each test. Regarding hypothesis (1), we found evidence for stable measures of Emotionality as infant rhesus macaques mature into juveniles for cooing, barking, and—to a lesser extent—lip-smacking in holding cage observations. The consistency of cooing was also stable in human intruder tests. The lack of consistency in other BBA-associated behaviors could be due to age-related growth changes, individual experience, development of contextual specificity, or social learning (e.g., via sociobehavioral conformity—whereby conformity refers to increasing behavioral homogeneity among individuals, here associated with development in a social group [Whiten & van de Waal, 2018]). We explore these possibilities in greater detail below and provide some evidence of potential interactions with the experience of repeated observation and one Activity-associated behavior: proportion of time locomoting in holding cage observations. Temperament ratings did not show consistency; we discuss whether this discontinuity might be due to some aspects of this particular procedure. Regarding hypothesis (2), we observed the emergence of sex differences in behavioral responses especially in our assessments at individuals’ second year, which are likely associated with developmental milestones. Finally, regarding hypothesis (3), we focused on hospitalizations as a particular life experience that might be expected to alter responses to BBA; though, we note that other life experiences may also influence behavioral responses to BBA (e.g., competition with kin or siblings, within-group agonism, interactions with human technicians). We found partial evidence for a potential association between prior hospitalizations and BBA-associated locomotion; though the causality of this association is challenging to disentangle.

### 4.1 | Consistency of behavioral measures

A subset of the BBA-relevant behaviors were associated with the expected BBA factors. We found that, for males, cooing, barking, and—to a lesser extent—lip-smacking remain reasonable indicators of emotionality into their second year of development. Females, however, did not have the same level of consistency in their behavioral responses. Our work shows comparability with test–retest correlations obtained in the literature on human personality development (Caspi et al., 2005) and general behavioral repeatability estimates (Bell et al., 2009). Older individuals showed greater stability between the rates of behaviors, despite a larger period of time between the second and third sampling periods, relative to the first two sampling periods. We found discrepancies in repeatability between ratings, human intruder, and observational methods, which is surprising given expectations from the human literature (Caspi et al., 2005) that assessment methods should not differ in stability.

Observed discrepancies from the BBA factor scores in the experimental results may be partially attributed to age-related shifts in the expression of behaviors, shifts due to personal experiences, or learned contextual specificity in the use of particular behaviors and sociobehavioral conformity. We examined these possibilities via the consistency of behavioral measures. The BBA factors may, additionally, undergo growth related changes—which we examined via BBA factor consistency. These changes, however, are concurrent with sex differences that emerge as these individuals aged. Below, we outline the evidence for each of these points, both from our study and the relevant literature.

We generally found that younger infants (3–4 mos.) exhibited more behavioral changes (e.g., hyperactivity) in the holding cage observations and human intruder tests (Supporting Information: Figures S1 & S2), relative to later stages of development. These age- related shifts may be due to reduced responsiveness to the testing conditions due to habituation, sociobehavioral conformity from shared housing conditions, or learned fear responses. Additionally, individuals may have developed greater specificity in the context in which behaviors are expressed. During the first 2 years infants undergo rapid development in neural regions associated with discriminating between social contexts (Azzi et al., 2012; Hodel, 2018). Parsing out these different mechanisms of change is challenging in this study, but an important point of discussion in determining why some behaviors were no longer associated with particular factors or why we did not find consistency across development for these measures.

Despite the continued associations with Emotionality in males, we observed age-related declines in the expression of cooing vocalizations that parallel the findings of Kalin and Shelton (1998), who measured these vocalizations at 4, 8, and 12 months of age in infant macaques in response to being isolated and to a human intruder. Similar to this study, vocalizations of cooing steadily decreased as the infants aged and showed high Spearman’s rank correlations (0.41–0.76) across sample events. Kalin and Shelton (1998) describe an interaction between ontological changes and contextual shifts for when cooing is provoked; older individuals were posited to shift to contextually specific use of barks or threats. In our results, however, coos, threats, and barks all decreased from the original BBA to the 1-year replicate. Cooing, therefore, is likely to be undergoing an age-associated change, but because the later samples in our study replicate the same challenge, it remains unclear if there is a component of habituation.

We acknowledge that some behavioral changes might be due to a reduced effectiveness in the behavioral response itself. For example, decreases in the proportion of hanging in Y2 might be attributed to an increase in body size relative to cage size that could limit the viability of hanging as a response. Hanging from the side of the cage may be perceived as a more effective strategy for dealing with the unfamiliar situation when an infant is smaller, but an increased body size likely makes this strategy less feasible. Whether cages scaled to the body size of the subject would promote more hanging is unclear, as we did not alter holding cage size across development. Adults in the large outdoor housing units utilize hanging as a viable method of escape, though with less frequency than running terrestrially. Irrespective of the reason for the decline, hanging is unlikely to be relevant throughout development, but may be a measure of interest for Activity in Y1. Similarly, male rates of exploration may be a reliable measure for Activity in Y1. By Y2, however, many individuals spent a majority of their time sitting in the cage, instead of hanging; as with hanging, this change may be due to cage size or habituation to the experimental conditions.

### 4.2 | Influence of experience

As an indirect proxy for the influence of experience, we examined whether there were associations between the relevant BBA behaviors and hospitalization events. Repeated exposure to the capture process and housing associated with hospitalizations may result in desensitization to aspects of the BBA (e.g., social separation and indoor laboratory housing management practices). In five of the eight comparisons, hospitalization events were correlated with proportion of time locomoting during the holding cage observations—most evident in d1 for males. In the present study, locomote was coded as “Directed movement from one location to another” (Golub et al., 2009, p. 50); locomotor function in this context is unclear. A parallel measure to locomoting, however, might be pacing, which has been analyzed as a motor stereotypy (Gottlieb et al., 2013). Gottlieb et al. (2013) reported that cumulative relocation events increased stereotypies—which may be an explanation for why we observed higher locomotion in animals that had been hospitalized more frequently. This relationship, however, was not found in an earlier study (Lutz et al., 2003) and recent work, using an aggregate measure of stereotypy, found the opposite association (Polanco, 2021).

Alternatively, pacing has been proposed to be a proactive coping strategy (Ferreira et al., 2016) that covaries with locomotion. If so, individuals that have been hospitalized more frequently might have learned more effective coping behaviors. For instance, coping styles are distinct and individually consistent with stress response strategies, which have been quantified in wild primates (Koolhaas et al., 1999; Pritchard & Palombit, 2022) and which, in mice and rats, covary with aggression (Koolhaas et al., 1999). Indeed, BBA scores have been used to quantify subjects’ behavioral inhibition (Capitanio et al., 2022), which is a core component of coping style variation (Koolhaas et al., 1999). Individuals that scored low on Activity and Emotionality would be identified as having increased inhibition (Capitanio et al., 2022; Capitanio, 2018). Interestingly our association between hospitalization events and locomotion parallel those from Simpson, Sclafani, et al. (2019). Increased handling time from human caregivers across the first 2 weeks of life, in captive rhesus macaques, was associated with increased locomotion during a novel environment test. Simpson, Sclafani, et al. (2019) suggested that increased handling induced a decrease in infant sensitivity to the stress of a novel environment.

Finally, we acknowledge that subjects might have underlying personality traits that covary with or contribute to the risk of hospitalization. For instance, prior work has shown that adult subjects that were more frequently observed alone in a social group had higher trauma rates relative to more social individuals, independent of rank (Myers et al., 2021); similarly, subjects with lower ratings on the macaque Social Responsiveness Scale-Revised had higher rates of trauma, relative to high rated subjects (Myers et al., 2021). Elfenbein et al. (2016) also demonstrated that higher scores on the BBA Nervous temperament factor were associated with increased occurrences of diarrhea, relative to individuals with lower ratings. This effect, however, was more pronounced in nursery- versus outdoor-reared subjects. Due to the number of our subjects and interest in lifetime experience as a mediating effect, we pooled all hospitalization records irrespective of the reason for hospitalization. Our findings, however, justify future investigation into the coupled dynamics and causal relationships of lifetime experiences with the development of personality-like traits.

### 4.3 | BBA factor consistency

An aggregate understanding of behavioral consistency for all of the factor-associated behaviors can give us insight into developmental shifts from temperament-like traits to personality-like traits. We observed greater stability in Emotionality-associated behaviors, but reduced stability in Activity-associated behaviors in both the human intruder test and holding cage observations. These results parallel outcomes from Stevenson-Hinde et al. (1980) who annually isolated three survey-rated components from rhesus macaques over 4 years. Excitable and Sociable components were not consistent, but Confident component scores were consistent across years. Excitable was composed of movement items (active, slow, not easily disturbed [equable]) and may be analogous to our Activity factor. Their Confident component (fearful, aggressive, apprehensive, confident) could be associated with the expression of Emotionality-like behaviors, which were stable in this study.

Further parallel evidence comes from Sussman and Ha (2011) who used responses to novel costumes worn by handlers. Sussman and Ha (2011) reported declines in mean reactivity scores over the first 300 days of development, measured by irritability, consolability, and vocalizations. These outcomes are similar to our study’s age-related decreases in Emotionality-associated behaviors. Interestingly, Sussman and Ha (2011) observed a sigmoidal increase in scores on their boldness component, as measured by approach, contact, and exploration throughout the first 300 days of development. We did not see an increase in Activity during the relevant age brackets; Activity remained relatively unchanged in our study but may have already stabilized, as anticipated by the boldness component in Sussman and Ha (2011). Our human intruder test, however, showed decreases in proportions of time active and environmental exploration across the 2 years. We posit that this directional discrepancy between the two studies is attributable to differences in the testing design or taxonomic distinctions, rather than growth patterns.

Interestingly, Sussman and Ha (2011) found that the latent structure of their temperament components shifted as individuals aged. Dynamic approaches to fluctuating latent structures may need to be utilized to understand how underlying personality traits manifest differently over time. For instance, in humans, Activity is found to be a strong component of personality during childhood, being dubbed a sixth personality dimension (Soto & John, 2014). This dimension, however, has a nuanced development and has been posited to merge with dimensions of Extraversion and Conscientiousness as individuals reach adulthood (Soto & John, 2014; Soto& Tackett, 2015). How these differences manifest behaviorally is, to our knowledge, unclear. Compelling evidence for such changes in a nonhuman primate, however, comes from von Borell et al. (2016) who reported that free-ranging rhesus macaques progressively transitioned from resting and submissiveness, to friendly approaches and proximity between the first and third years of life. This is compelling because resting is a measure of a lack of physical activity, while the latter behaviors of friendly approaches and proximity can be viewed as a social aspect, associated with extraversion. Such changes, however, are difficult to extricate from social development itself. Furthermore, personality ratings on 55 infant rhesus macaques revealed a personality structure similar to factors isolated in studies on adult macaques providing some evidence for structural stability across development (Simpson, Robinson, et al., 2019). Future studies on the developmental expression of activity under controlled conditions are necessary, but such studies must also incorporate metrics of extraversion to appropriately examine this putative transition.

### 4.4 | Test reproducibility

Three tests in the BBA were repeated two-to-three times. Overall, holding cage observations showed the greater within individual consistency over the years, especially for the d1 observations. The d1 observations are associated with behavioral responsivity to the initial challenge of removal from their social unit, while the d2 responses seem to reflect more the animals’ adaptation to the test environment. Although the Y1 human intruder test results showed high consistency with the original BBA factor scores, the Y2 results were challenging to interpret due to a large absence of BBA-associated behaviors. Given the direct association of the human intruder with the subject, it seems likely that this change may be attributed to a learned response—though this assertion is challenging to test with the current data set. In males, the composite temperament scores as yearlings showed poor association with infant temperament scores. In females, only the Nervous yearling score showed a moderate association with Nervousness in infancy. These temporal distinctions might be due to minor shifts in item ratings as outlined by Stevenson-Hinde et al. (1980). To avoid this as a confounding factor across sample periods, Stevenson-Hinde et al. (1980) omitted items that had low loading values. Their survey, however, had 21 items loading on three components, as opposed to the 14 we have on four factors. Thus, this approach to shifts in item loadings was not feasible in the present study. Replications of the temperament rating paradigm using surveys with a greater number of items might facilitate greater flexibility via item removal. Furthermore, inclusion of a greater number of raters could facilitate greater insight into whether there were challenges in rating particular individuals or items, though research from humans suggests that variation in subjects’ age and gender can influence inter-rater reliability (Slobodskaya, 2021).

### 4.5 | Age-related changes and sex differences

In our study, both sexes had lower consistency between their BBA scores and their second-year behaviors, relative to their first-year behaviors. Males, however, showed more robust associations with BBA factor scores in their second-year behaviors, relative to females. This distinction importantly coincides with findings from free living rhesus macaques on Cayo Santiago where 2 years of age was nominated as an important time for behavioral change between the sexes (Kulik et al., 2015a, 2015b). In their isolation and human intruder tests, Kalin and Shelton (1998) did report that there were no sex differences for infants between 4 and 12 months of age. Together, these findings parallel the assertion that behavioral traits often show reduced consistency across developmental periods, as emphasized in a large multispecies review (Cabrera et al., 2021). The 2-year mark precedes sexual maturation in our population. These outcomes are similar to findings from von Borell et al. (2016) who reported sex-based divergence in a fearfulness factor emerging around the age of sexual maturation. Interestingly, studies on human personality development indicate that girls mature faster (Slobodskaya, 2021), which might account for the later inconsistency observed in female, but not male, 2- year-olds. As such, it seems plausible that measuring the same BBA factors into-and-after 2 years of age would require explicit consideration of sex differences—at least when relying on behaviors.

### 4.6 | Comparability to findings in human research

Clear parallels between our results and those from studies of human development are complicated by a variety of compounding factors. Human temperament and personality are typically measured and understood via independent factor scales assembled from ratings: studies on adults often assume a five-factor model, while studies in children lack a clear consensus regarding the number of factors (ranging from one-to-six [Rothbart, 2007; Slobodskaya, 2021; Soto & John, 2014; Zentner & Bates, 2008]). Furthermore, few cross-sectional studies exist (Caspi et al., 2005; Slobodskaya, 2021). A repeating conclusion, however, is that there are hierarchical dynamics across development for temperament-personality frameworks—such that children’s temperament factors are likely subsumed by or combine to form adult personality dimensions (Caspi et al., 2005; Slobodskaya, 2021; Soto & Tackett, 2015).

Validation of BBA factors in rhesus macaques and human temperament factors would be necessary before direct synthesis. Studies of adult rhesus macaque personality structure often find three-to-five factors (Capitanio, 1999; Maninger et al., 2003; Stevenson-Hinde et al., 1980; Weiss et al., 2011)—resolving the number of adult and infant personality factors across situations and developmental milestones would be paramount before drawing concrete parallels. Even so, among factors that showed at least partial consistency, we can posit that Emotionality likely parallels a human Negative Emotionality/Behavioral Inhibition factor, and Activity likely parallels Activity or Attention/Effortful Control, based on descriptions in Zentner and Bates (2008). In humans, greater Effortful Control can result in emotional restraint and increases as infants age, with a greater increase in girls (Kochanska et al., 2000); such a finding could parallel our finding of sex differences and reduced behavioral expression.

### 4.7 | Limitations

Our subset of subjects was less representative of the extremes of the BBA human intruder data set relative to the temperament ratings and holding cage tests, as evident by the standard deviations of our BBA factor scores. One of our factors, Displacement, had correlations with two behaviors, tooth grinding and yawning, that were driven by several outliers. Thus, our findings warrant future replications with larger sample sizes that are more representative of the variation in the full BBA infant data set. Our subjects were only from a large outdoor mixed-sex housing unit. This distinction is of relevance as rearing conditions have been showed to drive differences in BBA scores (Capitanio et al., 2006; Gottlieb et al., 2013). While rearing condition was uniform in our group, we acknowledge that there might be other aspects of experience that influence personality development. Thus, we included hospitalization events, but acknowledge that other factors we did not analyze here may also be influential—such as maternal style of rearing, mother’s rank, infant’s social bonds, or major life stressors.

Unfortunately, the development trajectory of the BBA factors, and personality traits writ-large, is underresearched and there are many uncertainties as to whether age-related changes in our behavioral measures were due a lack of scaling in the test design, habituation, social conformity, changes in contextual specificity for behaviors, or shifts in the underlying traits themselves. We emphasize, however, the uniqueness of this data set and the robust sample sizes from the original BBA data set as providing robust underlying validity, despite these uncertainties. Even so, we suggest that BBA-like protocols in older juveniles might warrant future consideration of age-relevant behaviors or factors—such as Positive Emotionality or Extraversion. Drawing upon a larger subject pool would facilitate building independent factor structures at each time point but introduces complexity of uniting these factor structures across samplings. Furthermore, the context or situation of BBA is constrained as a sustained challenge for the subject—this might limit which factors can be sampled during this time period (e.g., Neuroticism, Coping Style, or Reactivity-like traits); though, rehousing of any type would likely cause a similar methodological conundrum. Importantly, removal of the challenge of rehousing could facilitate measurement of Positive Emotionality-like traits.

### 4.8 | Conclusion

Several measures of Emotionality showed consistency across the three test periods for measures from both the holding cage observations and human intruder tests, especially in male infants. Activity may undergo more complex changes as a dimension of personality, an assertion that parallels findings in the human personality literature. Finally, sex differences may emerge as infant macaques age via the expression of particular behaviors, and there is likely contextual nuance in the use of these BBA factor-associated behaviors.

## Supplementary Material

Supinfo

## Figures and Tables

**TABLE 1:** Holding cage observation Spearman’s rank correlations between BBA scores (from infancy) and raw behavioral rates or proportions (from Years 1 and 2).

			Activity						Emotionality				
	
Sex	Day	Year	Hang proportion	Explore rate	Locomote proportion	Crouch rate	Drink rate	Eat rate	Lip-smack rate	Self-scratch rate	Threat rate	Bark rate	Coo rate
Female	D1	One	−0.23	0.22	0.20	0.07	−0.04	0.07	**0.40** *	−0.03	**−0.30**	0.16	**0.52** *
		Two	0.04	**0.53** **	0.17	0.08	−0.04	**−0.44** *	0.22	−0.09	−0.24	0.10	0.22
	D2	One	**−0.52** **	**0.49** *	−0.29	0.26	−0.20	0.03	**0.60** **	**0.56** **	0.15	**0.49** *	**0.40** *
		Two	0.02	−0.06	−0.02	−0.08	0.18	**−0.31**	0.17	0.10	−0.13	0.29	0.28
Male	D1	One	**−0.44**	**0.35**	0.16	0.16	0.06	−0.10	**0.63** **	0.02	**0.38**	**0.46** *	**0.76** **
		Two	−0.06	0.03	−0.07	−0.04	0.19	−0.01	0.25	0.14	0.04	**0.68** **	**0.52** *
	D2	One	−0.07	**0.32**	−0.07	−0.03	0.17	0.08	0.04	−0.21	**−0.45** *	−0.27	0.03
		Two	−0.08	0.18	0.15	0.08	0.08	**−0.42**	0.06	−0.20	−0.15	−0.21	0.09

*Note*: The table is divided by sex for each year, day, and BBA factor. Values ≥0.30 are in bold. We labeled unadjusted significance for coefficients ≥0.30.

* 0.05–0.01.

** <0.01.

**TABLE 2:** Human intruder test Spearman’s rank correlations between BBA scores (from infancy) and raw behavioral rates or proportions (from Years 1 and 2).

		Activity			Emotionality			Aggression			Displacement	
			
Sex	Year	Activity proportion	Explore rate	Cage Shake rate	Coo rate	Grimace rate	Self-Clasp rate	Barking rate	Threat rate	Vocalize (Other) rate	Tooth Grind rate	Yawn rate
Female	One	**0.46** *	**0.49** *	0.06	0.29	**0.34**	**0.53** **	**0.37**	**0.39**	−0.28	**0.58** **	**0.44** *
	Two	0.01	–	–	0.04	–	–	–	–	–	–	–
Male	One	0.06	0.08	0.07	**0.49** *	**0.43**	0.04	**0.66** **	−0.04	0.10	**0.61** **	0.29
	Two	0.26	–	–	**0.32**	–	–	–	–	–	–	–

*Note*: The table is divided by sex for each year and BBA factor. Note that many behaviors were not expressed during the human intruder tests in Y2. Values ≥0.30 are in bold. We labeled unadjusted significance for coefficients ≥0.30.

* 0.05–0.01.

** <0.01.

**TABLE 3:** Spearman’s rank correlations between Year 1 and Year 2 raw behavioral rates or proportions.

Holding cage observations									

		Activity			Emotionality				
			
Sex	Day	Hang proportion	Explore rate	Eat rate	Coo rate	Lip-smack rate	Self-scratch rate	Threat rate	Bark rate
Female	D1	−0.20	**0.46** *	0.18	**0.67** **	**0.45** *	**0.76** **	**0.45** *	**0.62** **
	D2	0.02	**0.42**	0.15	**0.50** *	0.20	0.02	**0.72** **	**0.64** **
Male	D1	0.22	**0.44**	**0.46** *	**0.71** **	**0.52** *	**0.65** **	−0.08	**0.37**
	D2	0.06	0.15	0.09	**0.54** *	**0.33**	0.19	**0.35**	**0.43**

Human intruder test		

Sex	Activity proportion	Coo rate
Female	0.09	0.04
Male	0.06	**0.54** *

*Note*: The table is divided by test, sex, and BBA factors. Values ≥ 0.30 are in bold. We labeled unadjusted significance for coefficients ≥0.30.

* 0.05–0.01.

** <0.01.

**TABLE 4:** Spearman’s rank correlations between hospitalization events and raw behavioral rates or proportions during holding cage observations.

			Activity						Emotionality				
	
Sex	Day	Year	Hang proportion	Locomote proportion	Crouch rate	Drink rate	Explore rate	Eat rate	Lip-smack rate	Self-scratch rate	Threat rate	Bark rate	Coo rate
Female	D1	One	−0.26	0.12	−0.23	0.28	0.15	**0.40** *	−0.17	−0.03	−0.04	−0.21	0.06
		Two	0.25	**0.38**	0.13	**0.68** **	0.21	−0.05	−0.12	0.20	0.09	−0.05	0.20
	D2	One	**0.31**	**0.38**	**−0.31**	−0.01	0.25	−0.17	−0.17	0.03	−0.02	−0.01	0.17
		Two	−0.04	0.07	0.21	−0.01	0.16	0.08	−0.14	−0.27	0.05	−0.23	−0.13
Male	D1	One	−0.06	**0.44**	−0.07	0.08	0.17	0.26	0.19	0.13	**0.38**	**0.44**	0.23
		Two	**0.59** **	**0.81** **	−0.11	**0.34**	**0.43** *	0.22	**0.65** **	**0.31**	0.15	**0.33**	**0.55** *
	D2	One	0.20	**0.46** *	0.02	**−0.34**	0.06	0.12	−0.05	**0.64** **	0.19	0.20	**0.38**
		Two	0.22	0.02	0.28	−0.19	−0.20	**0.33**	−0.08	0.29	−0.03	0.08	0.00

*Note*: The table is divided by sex, year, day, and BBA factors. Values ≥0.30 are in bold. We labeled unadjusted significance for coefficients ≥0.30.

* 0.05–0.01.

** <0.01.

## Data Availability

The data that support the findings of this study are openly available in DataDryad at https://doi.org/10.25338/B8ND28.

## Highlights

- We ran biobehavioral assessments (BBA) on 47 subjects, replicating BBA at ~3.4 months-, one year-, and two years-of-age to provide insight on the development of individual differences and the design of relevant assessments over time.
- Results supported that Emotionality-associated behaviors exhibited rank consistency over time, more so than for Activity and other BBA factors; sex and age differences were evident.
- The root causes of variability in BBA-associated behaviors are likely complex, and we provide evidence that frequent life experience events (hospitalizations) are associated with behaviors exhibited during replicated BBA.

## Abbreviations:

BBA: BioBehavioral Assessment
CNPRC: California National Primate Research Center
