# Supplementary material for: Repeatability of measures of behavioral organization over two years in captive infant rhesus macaques, Macaca mulatta
Source: Am J Primatol. Author manuscript; Available in PMC 2025 Apr 1. (PMC11010731; doi:10.1002/ajp.23591)
Supplement: Supinfo [file NIHMS1955241-supplement-Supinfo.docx]

**Supplementary Materials.**

**Repeatability of measures of behavioral organization over two years in captive infant rhesus macaques, *Macaca mulatta***

Alexander J Pritchard^a^, John P. Capitanio^a^, Laura Del Rosso^a^, Brenda McCowan^a^, Jessica Vandeleest^a^

^a^ California National Primate Research Center, University of California, Davis, California, USA

**Supplementary**

**
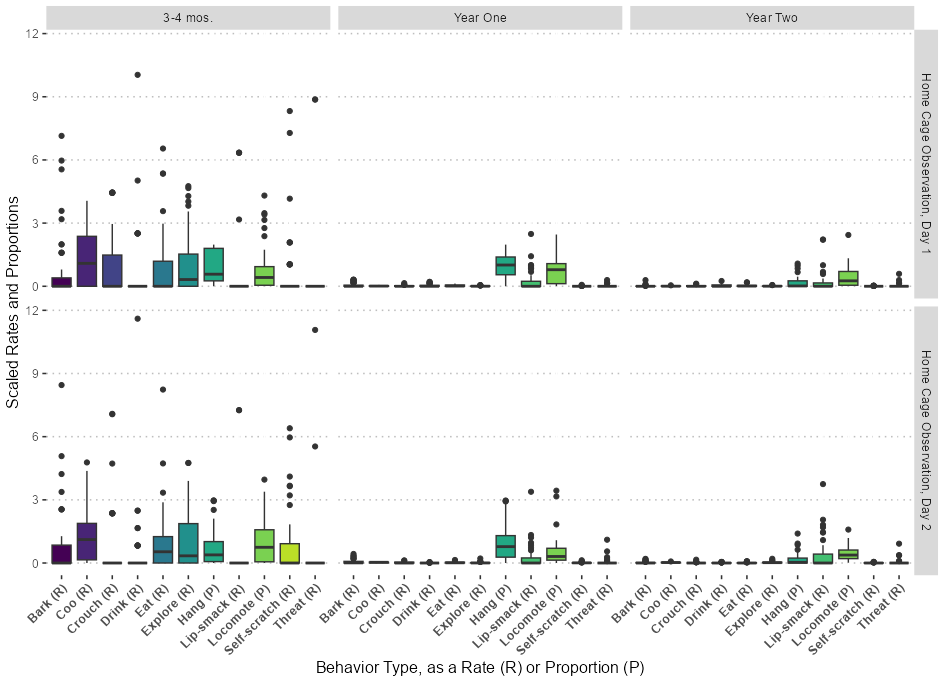
**

**Supplementary Figure 1.** Boxplots of changes in abundance of each type of BBA-associated behavior for holding cage observations on day 1 (top panel) and day 2 (bottom panel) across years (columns). Behaviors were scaled within each type for plotting; x-axis represents each behavior type (appended with an ‘(R)’ or ‘(P)’ for rates and proportions, respectively) and the y-axis shows the scaled rates or proportions for each behavior.

**
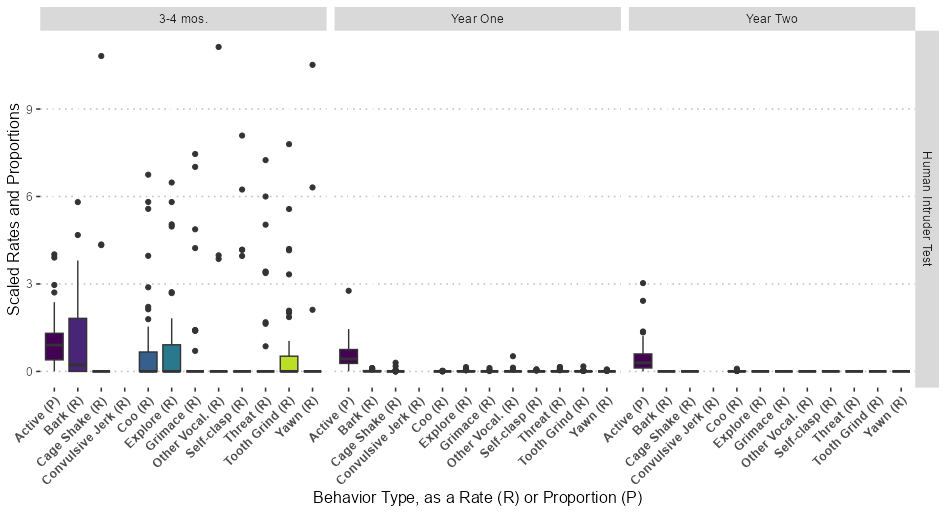
Supplementary Figure 2.** Boxplots of changes in abundance of each type of BBA-associated behavior for the human intruder test across years (columns). Behaviors were scaled within each type for plotting; x-axis represents each behavior type (appended with an ‘(R)’ or ‘(P)’ for rates and proportions, respectively) and the y-axis shows the scaled rates or proportions for each behavior.

**
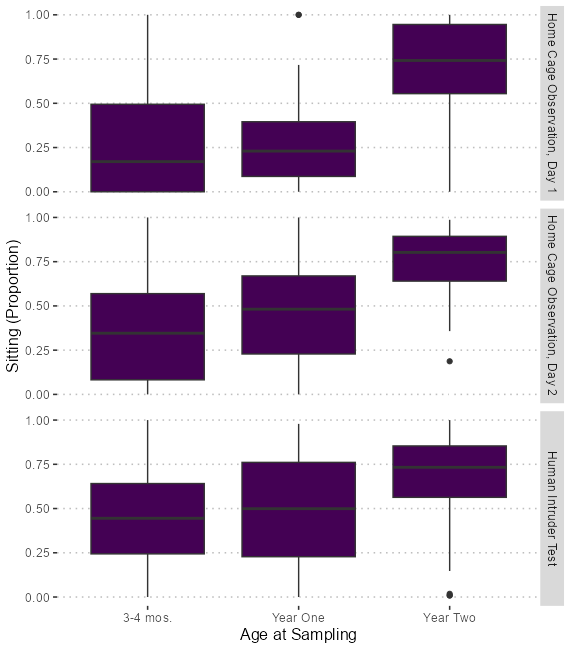
**

**Supplementary Figure 3.** Boxplots of proportion of time sitting (y-axis) across year (x-axis) for each of the BBA tests (row panels). Central lines in each box represent medians, boxes extend to the 25^th^ and 75^th^ percentiles, with whiskers representative bound to 1.5*IQR; outliers from the whiskers are represented as points.


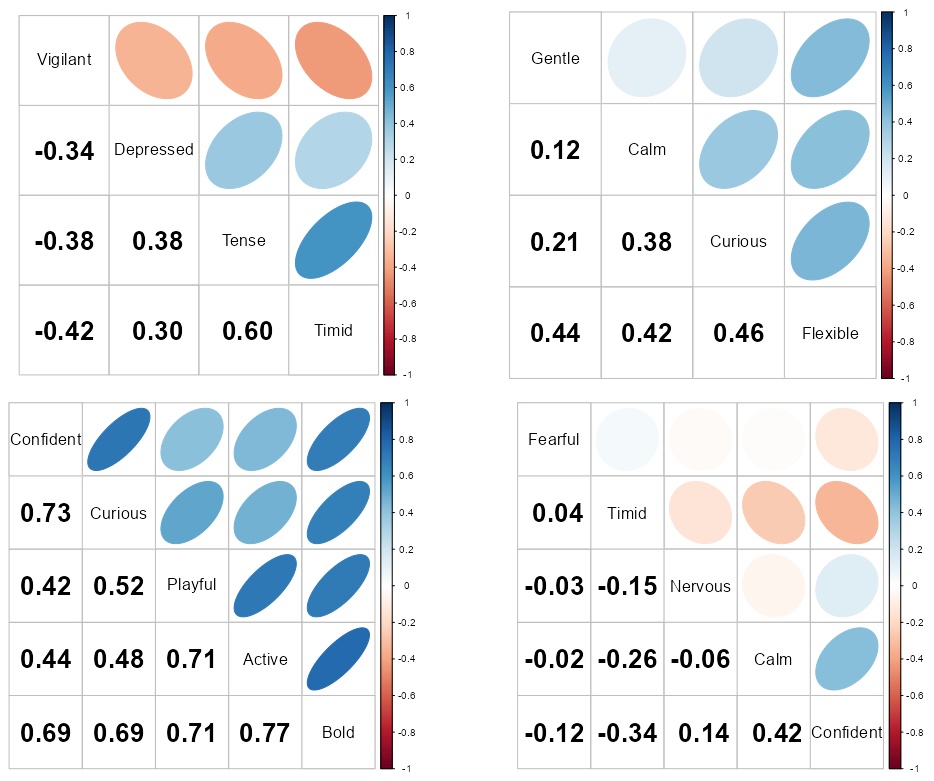


**Supplementary Figure 4.** Cross correlations between Likert rated temperament items within each expected factor for year 1 items ratings. Clockwise from the upper-left: Vigilant, Gentle, Nervous, and Confident. In the upper half of each matrix, color and shape of ellipse is representative of direction and strength of the correlation. In the lower half of each matrix, numerical values for each cross correlation.


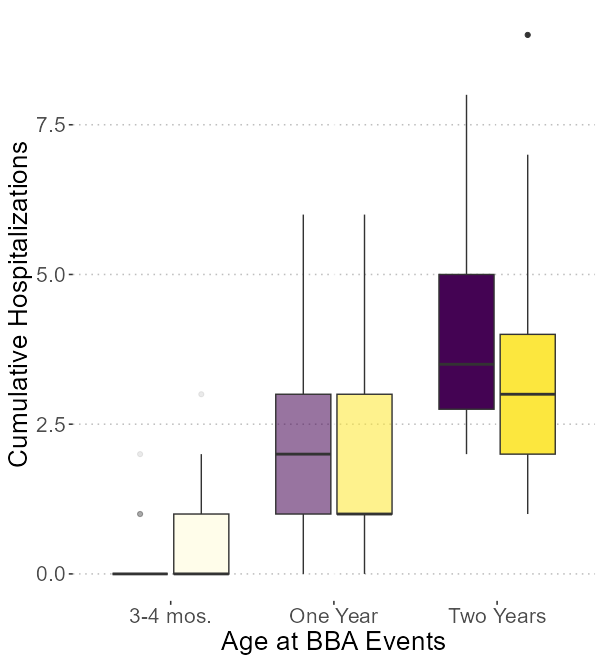

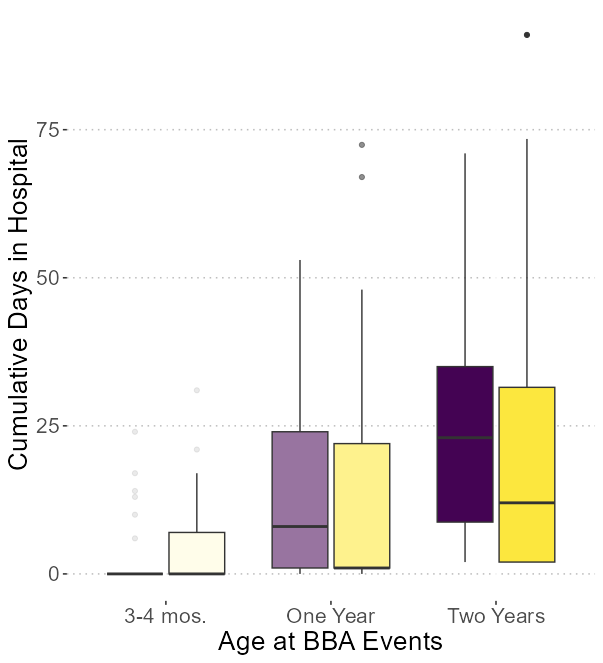


**Supplementary Figure 5.** Box plots of cumulative hospitalization events (left) or cumulative days in hospital (right) by the age at each BBA event (x-axis). Fill color represents males (yellow) and females (purple) with alpha emphasizing year. Central lines in each box represent medians, boxes extend to the 25^th^ and 75^th^ percentiles, with whiskers representative bound to 1.5*IQR; outliers from the whiskers are represented as points.

**Supplementary Table 1.** Spearman’s rank correlations between hospitalization events and raw behavioral rates or proportions during human intruder tests. The table is divided by sex, year, day, and BBA factors. Values ≥0.30 are in bold. We labelled unadjusted significance for coefficients ≥0.30; * = 0.05-0.01; ** < 0.01.

|  |  | **Activity** | | | **Emotionality** | | | **Aggression** | | | **Displacement** | |
| --- | --- | --- | --- | --- | --- | --- | --- | --- | --- | --- | --- | --- |
| Sex | Year | Activity Proportion | Explore Rate | Cage Shake Rate | Coo Rate | Grimace Rate | Self-Clasp Rate | Barking Rate | Threat Rate | Vocalize (Other) Rate | Tooth Grind Rate | Yawn Rate |
| Female | One | -0.19 | **-0.31** | 0.13 | -0.06 | -0.01 | -0.13 | 0.00 | -0.01 | -0.01 | -0.11 | 0.23 |
|  | Two | 0.25 | **-** | **-** | **0.51**** | **-** | **-** | **-** | **-** | **-** | **-** | **-** |
| Male | One | **0.50*** | 0.13 | 0.14 | -0.13 | 0.25 | **0.38** | **0.37** | -0.11 | 0.04 | 0.01 | -0.13 |
|  | Two | 0.28 | **-** | **-** | 0.36 | **-** | **-** | **-** | **-** | **-** | **-** | **-** |

**Supplementary Table 2.** Spearman’s rank correlations between hospitalization events and year 1 composite temperament scores from ratings. The table is divided by sex and BBA factors. Values ≥0.30 are in bold. We labelled unadjusted significance for coefficients ≥0.30; * = 0.05-0.01.

| Sex | **Confident** | **Gentle** | **Nervous** | **Vigilant** |
| --- | --- | --- | --- | --- |
| Female | 0.26 | 0.01 | -0.06 | -0.11 |
| Male | **0.45*** | 0.08 | **-0.42** | **0.50*** |

**Supplementary Table 3.** Spearman’s rank correlations between year 1 and year 2 raw behavioral rates or proportions that did not exceed |0.3| rank correlations with BBA scores. The table is divided by sex and BBA factors. Values ≥0.30 are in bold. We labelled unadjusted significance for coefficients ≥0.30; * = 0.05-0.01; ** < 0.01.

|  |  | **Activity** | | |
| --- | --- | --- | --- | --- |
| Sex | Day | Locomote  Proportion | Crouch Rate | Drink Rate |
| Female | D1 | **0.59**** | **0.43*** | 0.24 |
|  | D2 | 0.04 | 0.06 | -0.03 |
| Male | D1 | **0.75**** | **0.48*** | **0.67**** |
|  | D2 | **0.38** | **0.35** | -0.17 |
